# Extra Virgin Olive Oil Polyphenols Promote Cholesterol Efflux and Improve HDL Functionality

**DOI:** 10.1155/2015/208062

**Published:** 2015-10-01

**Authors:** Hicham Berrougui, Souad Ikhlef, Abdelouahed Khalil

**Affiliations:** ^1^Department of Medicine, Geriatrics Service, Faculty of Medicine and Biological Sciences, University of Sherbrooke, 3001 12e Avenue Nord, Sherbrooke, QC, Canada J1H 5N4; ^2^Department of Biology, Polydisciplinary Faculty, University Sultan Moulay Slimane, BP 592, 23000 Beni Mellal, Morocco

## Abstract

Results of the present work give evidence from the beneficial role of extra virgin olive of oil (EVOO) consumption towards oxidative stress and cardiovascular diseases. Polyphenols contained in EVOO are responsible for inhibiting lipoproteins oxidative damages and promoting reverse cholesterol transport process via ABCA1 pathway.

## 1. Introduction

Coronary heart disease (CHD) is the main cause of mortality in the Western world. The oxidation of low-density lipoproteins (LDL) is an early event in the development of atherosclerosis, the underlying cause of CHD [1]. Oxidized LDL are not recognized by the LDL-receptor Apo (B/E) but are taken up by macrophages in a nonregulated manner through the scavenger-receptor pathway, which leads to the formation of foam cells, the hallmark of arteriosclerotic lesions [1].

Macrophage-specific reverse cholesterol transport (RCT) is thought to be one of the most important HDL-mediated cardioprotective mechanisms. RCT is the process by which cholesterol in peripheral cells is effluxed onto circulating HDL and is transported back to the liver for excretion in bile and feces [2, 3]. The promotion of RCT is considered a major antiatherogenic function of HDL [4, 5]. The efflux of cholesterol from cells to HDL is the first and rate-limiting step of RCT [6]. Two major macrophages cholesterol efflux pathways have been described: SR-BI receptor-mediated cholesterol efflux and ABCA1/ABCG1-mediated cholesterol efflux. ABCA1 promotes the efflux of phospholipids and cholesterol to lipid-poor apo-AI via a process that involves the direct binding of apo-AI to the ABCA1 transporter, whereas ABCG1 and SR-BI are key mediators of macrophage cholesterol efflux to mature HDL [7]. Evidence from a recent study indicates that the inflammatory process induces changes in HDL composition and metabolism that impair RCT [8]. Interestingly, we recently showed that RCT is also impaired with aging, especially by changes to the ABCA1-mediated cholesterol efflux pathway [9, 10].

Polyphenol-rich vegetable oils and monounsaturated fatty acids provide protection against an array of human diseases such as cancer, atherosclerosis, and CVD, including those involving the central nervous system. Olive oil, which is known for its healthful properties, which are often attributed to its high monounsaturated fatty acid content, including oleic acid (18:1 n-9), is a prominent member of the family of polyphenol- and monounsaturated fatty acid-rich oils. However, olive oil, unlike other vegetable oils, contains high amounts of several micronutrient constituents, including polyphenolic compounds (100–1000 mg/Kg) such as hydroxytyrosol, tyrosol, and oleuropein [11].* In vitro* and* in vivo* human and animal studies have shown that EVOO reduces blood pressure [12], improves the lipid profile by increasing HDL-cholesterol and reducing LDL-cholesterol and triglyceride levels [13–15], reduces oxidative stress, and inhibits human lipoprotein oxidation, making LDL, for instance, less atherogenic [16, 17]. Olive oil dietary supplementation decreases the levels of high inflammatory and endothelial dysfunction markers in the serum. Experimental and clinical studies have shown that olive oil downregulates vascular cell adhesion molecule-1 (VCAM-1), human soluble intercellular adhesion molecule-1 (sICAM-1), and E-selectin expression in the endothelium [18] and decreases plasma levels of sICAM-1, soluble E-selectin, interleukin-6 (IL-6), and high-sensitive C-reactive protein (CRP) in high-risk patients [19].

The beneficial effects of polyphenols appear to be mediated via a plethora of biochemical pathways and signaling mechanisms that act either independently or synergistically. In the present study, we investigated the atheroprotective effect of the phenolic compounds in EVOO on cholesterol efflux and on oxidative stress damage in healthy subjects.

## 2. Methods

### 2.1. Subjects

Twenty-four healthy volunteers (30.92 ± 2.55 years) with normal serum lipid profiles and blood pressure were recruited. They were all nonsmokers and were not taking any medication, including lipid-lowering treatments or oral antioxidants. None of the female subjects was undergoing estrogen replacement therapy for menopause. None of the participants showed clinical signs of inflammation, obesity, or diabetes. The physical and biochemical parameters of the participants are presented in Table 1. The Ethics Committee of the Sherbrooke Geriatric University Institute approved the study, and all subjects provided written informed consent before being enrolled.

### 2.2. Phytochemistry

The phenolic compounds were extracted from EVOO using the method of Pirisi et al. [20]. Briefly, EVOO was mixed with* n*-hexane and methanol/water and was stirred in a vortex apparatus overnight at 4°C. The mixture was then centrifuged, and the hydroalcoholic solution was washed with* n*-hexane and then lyophilized overnight.

### 2.3. Lipoprotein Isolation

Fasting human plasma was collected in heparin tubes, and the HDL was immediately isolated using the method of Sattler et al. [21]. The isolated lipoproteins were dialyzed overnight at 4°C against 10^−2 ^M sodium phosphate buffer (pH 7.0). The protein concentrations were measured using commercial assay kits (Bio-Rad, Canada) using the manufacture's protocol.

### 2.4. Lipoprotein Enrichment with EVOO and EVOO-PC

Human plasma was incubated overnight with slight agitation at 4°C in the presence of EVOO (0.2 mg/mL of plasma) or EVOO-PC (1.76 mg/mL of plasma). The LDL and HDL were then isolated as described above.

### 2.5. Copper-Mediated Lipoprotein Oxidation

The lipoproteins were peroxidized as previously described using transition metal ions as oxidizing agents [22]. Briefly, control, EVOO, and EVOO-PC-enriched lipoproteins [(LDL 100 *μ*g/mL) or (HDL 200 *μ*g/mL)] were suspended in 10 mM sodium phosphate buffer (pH 7) and were incubated for 0 to 4 h at 37°C in the presence of 10 *μ*M cupric sulfate. The oxidation reaction was stopped by adding EDTA. Lipid peroxide formation was assessed by monitoring conjugated diene formation at 234 nm.

### 2.6. Cell Cultures

Human THP-1 monocytes and J774 macrophages were cultured in RPMI 1640 and DMEM medium, respectively. The media were supplemented with 10% heat-inactivated FBS, 50 mM 2-*β*-mercaptoethanol (only for THP-1), 2 mM L-glutamine, 5 mg/mL of glucose, and 100 U/mL of penicillin. The differentiation of the THP-1 monocytes into macrophages was induced by culturing the monocytes in the presence of 100 *μ*M PMA for 96 h.

### 2.7. Cholesterol Efflux Measurements

THP-1-derived macrophages and J774 macrophages were incubated in fresh growth medium containing 0.2 *μ*Ci/mL [^3^H]-cholesterol for 48 h or 1 *μ*Ci/mL [^3^H]-cholesterol for 24 h, respectively. The loaded cells were washed, equilibrated in serum-free medium containing 1% BSA for 12 h, washed again, and subjected to various treatments. The THP-1-derived macrophages were incubated for 24 h with (1) HDL-free medium, (2) HDL (50 *μ*g/mL), (3) EVOO-enriched HDL (OO-HDL), or (4) EVOO-PC-enriched HDL (PC-HDL).

[^3^H]-Cholesterol loaded THP-1-derived macrophages were subjected to oxidative stress by incubating them with 0.2 mM iron/ascorbate (Fe/Asc) in the absence or presence of EVOO-PC (320 *μ*g/mL) for 6 h. They were then incubated with HDL for 24 h to assess cholesterol efflux under various conditions.

The effect of EVOO-PC on ABCA1-mediated cholesterol efflux was assessed using J774 macrophages. [^3^H]-Cholesterol-loaded J774 macrophages were incubated for 12 h with 0 to 320 *μ*g/mL of EVOO-PC to generate ABCA1-enriched cells or with 300 *μ*M 8-Br-cAMP (positive control) to stimulate ABCA1 gene transcription and surface protein expression. The J7774 macrophages were then incubated with 25 *μ*g/mL of apo-AI for 4 h.

To better understand the mechanism of EVOO-PC-mediated cholesterol efflux, we studied the effect of the two major phenolic compounds in EVOO (tyrosol and hydroxytyrosol) on ABCA1-mediated cholesterol efflux. [^3^H]-Cholesterol-loaded J774 macrophages were incubated for 12 h with 0 to 25 *μ*M tyrosol or hydroxytyrosol to generate ABCA1-enriched cells and were then incubated with 25 *μ*g/mL of apo-AI for 4 h. 8-Br-cAMP was used as a positive control.

Cholesterol efflux was determined by liquid scintillation counting, and the percent of radiolabeled cholesterol released (percent cholesterol efflux) was calculated using the following formula: (cpm in the medium/[cpm in the cells + medium]) × 100.

### 2.8. Western Blot Analyses

ABCA1 protein expression in J774 macrophages was studied by incubating them for 12 h with 0 to 320 mg/mL of EVOO-PC or 5 or 10 *μ*M hydroxytyrosol or tyrosol. The proteins (20 *μ*g) were separated by electrophoresis on 10% acrylamide gels and were transferred to polyvinylidene difluoride (PVDF) membranes. The membranes were blocked with 5% milk in PBS/Tween 20 and were incubated with primary antibodies (anti-ABCA1) and then with specific IgG-HRP-conjugated secondary antibodies. *β*-actin was used as a control. The protein bands were detected using an enhanced chemiluminescence reagent (ECL) [10].

### 2.9. Statistical Analysis

Values are expressed as means ± SEM. A one-way analysis of variance (ANOVA) was used for multiple comparisons. A linear regression analysis was used to assess the association between two continuous variables. All statistical analyses were performed using GraphPad Prism-5 software.

## 3. Results

### 3.1. Effect of Extra Virgin Olive Oil and EVOO Phenolic Compound Extracts on Lipoprotein Oxidation

The concentration of total phenolic compounds (41.9 mM; gallic acid equivalent) was estimated using the Folin-Ciocalteu method.

The peroxidation by CuSO_4_ of the polyunsaturated fatty acids (PUFA) in HDL and LDL was assessed by the formation of conjugated dienes. The peroxidation kinetics showed that the lag phase of LDL was longer than that of HDL. The lag phase was followed by the propagation and termination phases.

**Figure 1:**
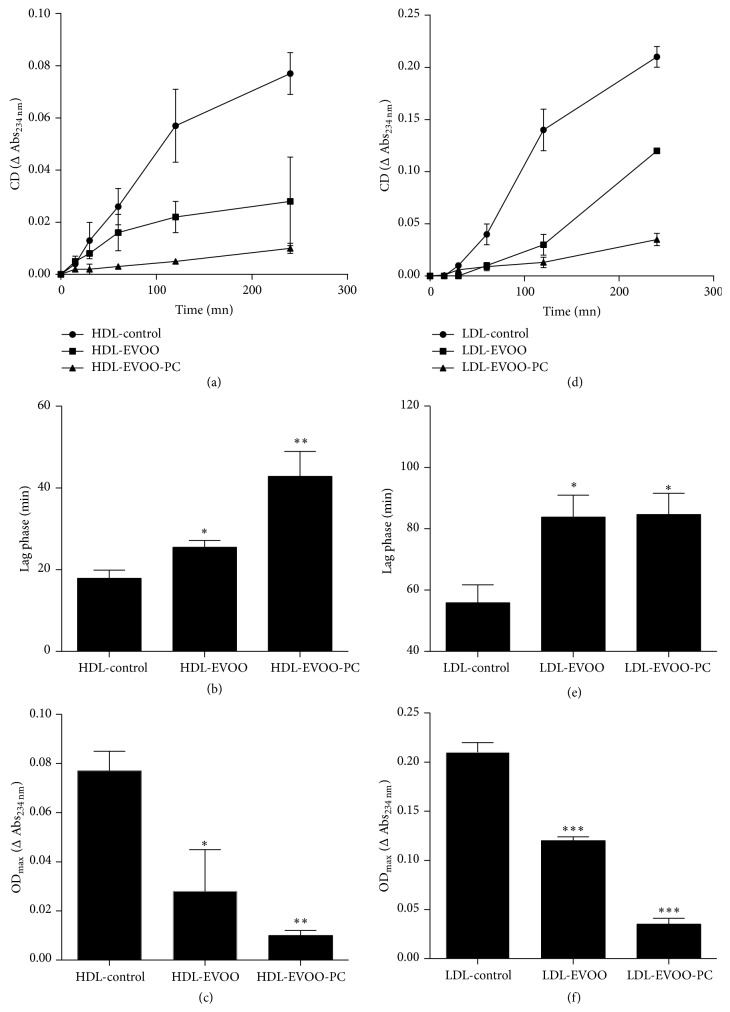
EVOO-PC enrichment decreases the oxidizability of lipoproteins. Plasma was incubated with EVOO or EVOO-PC prior to isolating HDL and LDL. HDL and LDL pretreated with EVOO or EVOO-PC as well as untreated controls were oxidized by incubation with copper ions for 4 h. The resistance to lipid peroxidation and the oxidizability of HDL and LDL were monitored by determining the lag phase (a, b, d, e) and by measuring conjugated diene formation (OD_max_), respectively (c, f). Results are expressed as the means ± SEM of three independent experiments. ^*∗*^ *p* < 0.05, ^*∗∗*^ *p* < 0.01, and ^*∗∗∗*^ *p* < 0.001.

Our results showed that plasma LDL and HDL that had been pretreated with EVOO or EVOO-PC were less oxidizible and were much more resistant to lipid peroxidation than untreated (control) plasma LDL and HDL as shown by the significant increase in the lag phase and the decrease in conjugated diene formation in EVOO-PC- and EVOO-treated lipoproteins (Figures 1(b) and 1(e)). The enrichement of lipoproteins with EVOO-PC or EVOO increased the lag phase 1.42- (*p* < 0.05) and 2.39-fold (*p* < 0.01) for HDL and 1.51- and 1.50-fold (*p* < 0.05) for LDL, respectively, compared to the control (Figures 1(b) and 1(e)). On the other hand, the enrichment of HDL and LDL with EVOO or EVOO-PC reduced conjugated diene formation (OD_max_) 4.53- (*p* < 0.05) and 7.71-fold (*p* < 0.01) for HDL and 1.75- (*p* < 0.001) and 14.58-fold (*p* < 0.0001) for LDL, respectively, compared to the control (Figures 1(c) and 1(f)).

### 3.2. Effect of Phenolic Compounds on Reverse Cholesterol Transport

**Figure 2:**
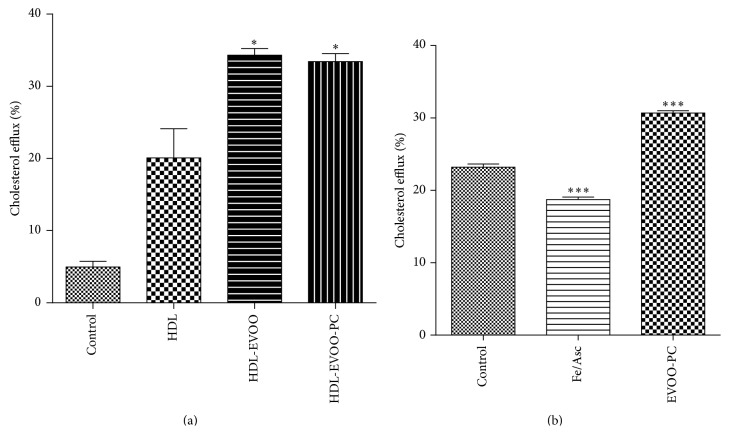
EVOO-PC protects macrophages against oxidation and promotes HDL-mediated cholesterol efflux. (a) THP-1-derived macrophages were loaded with [^3^H]-cholesterol (2 *μ*Ci/mL) for 24 h. The cells were then washed, equilibrated, and incubated for a further 24 h with 50 *μ*g/mL of HDL-free medium, HDL, EVOO-enriched HDL (EVOO-HDL), or EVOO-PC-enriched HDL (EVOO-PC-HDL). (b) The macrophages were stressed with 0.2 mM Fe/Asc, and cholesterol efflux was assessed using 50 *μ*g/mL of HDL. Results are expressed as the means ± SEM of at least three independent experiments. ^*∗*^ *p* < 0.05, ^*∗∗*^ *p* < 0.001, and ^*∗∗∗*^ *p* < 0.001.

To determine the effect of phenolic compounds on RCT, cholesterol efflux was measured. Incubating ^3^H-cholesterol-loaded THP-1-derived macrophages for 24 h with EVOO or EVOO-PC enhanced cholesterol efflux by 41.5% and 39.93% (*p* < 0.05), respectively, compared to the control (Figure 2(a)).

Oxidative damage to macrophages impairs cholesterol efflux, as shown by the decrease in ABCA1 protein expression induced by Fe/Asc [23]. We thus investigated the effect of EVOO-PC on the capacity of HDL to mediate cholesterol efflux in THP-1-derived macrophages under oxidative stress induced by Fe/Asc. HDL-mediated cholesterol efflux was significantly impaired under oxidative stress conditions whereas the effect was much lower when the macrophages were pretreated with 320 *μ*g/mL of EVOO-PC (*p* < 0.001) (Figure 2(b)).

**Figure 3:**
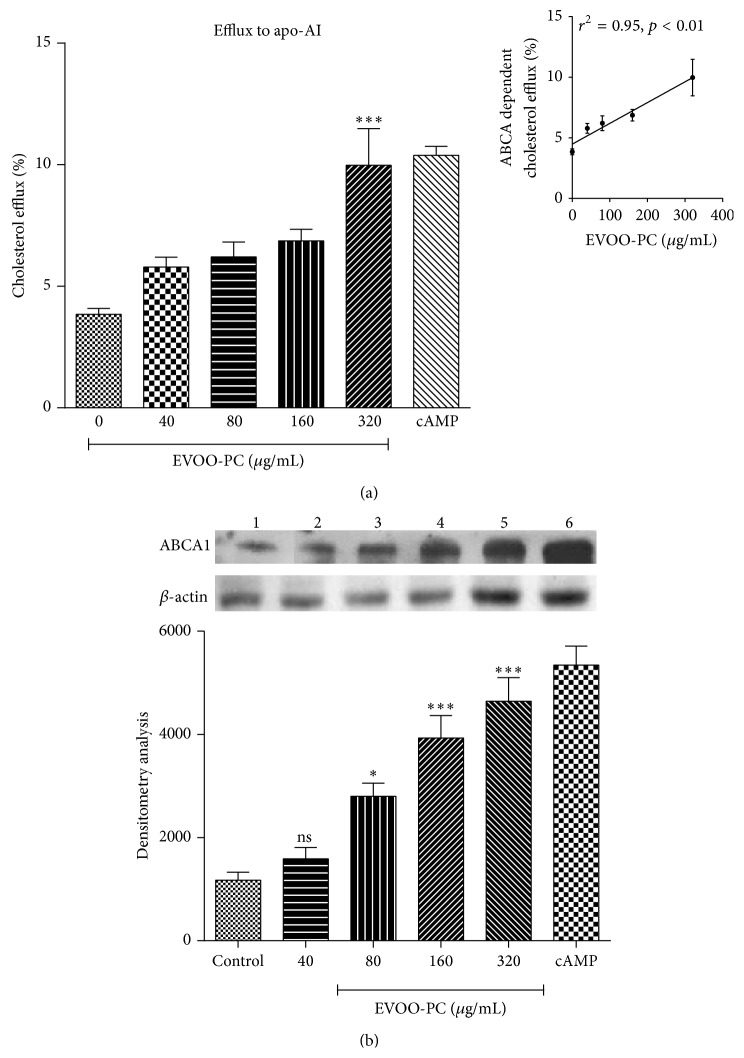
EVOO-PC increases ABCA1 protein expression and enhances apoA-I-mediated cholesterol efflux. (a) [^3^H]-Cholesterol-loaded J774 macrophages were incubated for 12 h with various concentrations of EVOO-PC (0 to 320 *μ*g/mL) or with cAMP (positive control) to generate ABCA1-enriched cells, which were incubated with 25 *μ*g/mL of apo-AI for 4 h. The small upper panel shows the positive correlation between EVOO-PC concentrations and ABCA1-dependent cholesterol efflux. (b) ABCA1 protein expression after incubating J774 macrophages with increasing concentrations of EVOO-PC as determined by densitometric analyses of protein bands on PVDF membranes. Results are expressed as the means ± SEM of at least three independent experiments. ^*∗*^ *p* < 0.05, ^*∗∗*^ *p* < 0.001, and ^*∗∗∗*^ *p* < 0.001.

To better understand the mechanism by which EVOO-PC enhances HDL-mediated cholesterol efflux, we investigated the effect of EVOO-PC on ABCA1-dependent cholesterol efflux from J774 macrophages. ^3^H-Cholesterol-loaded J774 macrophages were incubated with apoA-1 in the absence of cAMP for 4 h (time range for measuring cholesterol efflux via the ABCA1 pathway). We observed little cholesterol efflux in the absence of cAMP. However, when the macrophages were preincubated overnight with 0 to 320 *μ*g/mL of EVOO-PC or with a cAMP-analogue to induce ABCA1 protein expression and then with 25 *μ*g/mL of apoA-1 for 4 h, we observed a significant EVOO-PC concentration-dependent increase in cholesterol efflux (*r* ^2^ = 0.95, *p* < 0.01) (Figure 3(a)).

To investigate the mechanism by which EVOO-PC induces the increase in cholesterol efflux from J774 macrophages to apoA-1, we performed Western blot analyses to measure ABCA1 protein expression on J774 macrophages incubated with EVOO-PC. We observed an EVOO-PC concentration-dependent increase in ABCA1 protein expression in J774 macrophages incubated with EVOO-PC (Figure 3(b)).

**Figure 4:**
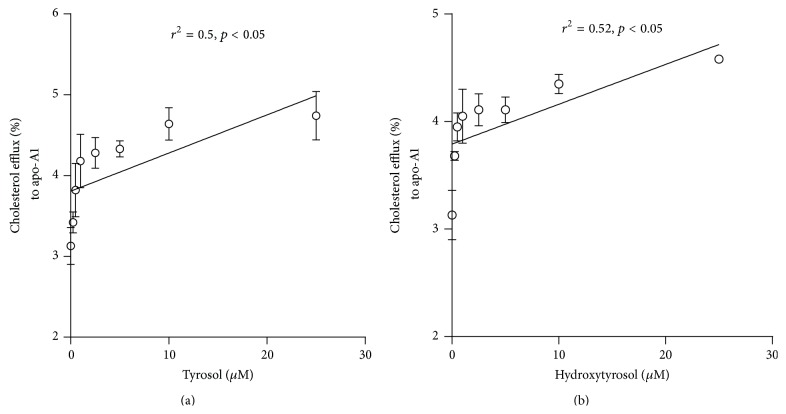
Tyrosol and hydroxytyrosol increase ABCA1 protein expression and enhance apoA-I-mediated cholesterol efflux. [^3^H]-Cholesterol-loaded J774 macrophages were incubated for 12 h with different concentrations (0 to 25 *μ*M) of tyrosol (a) or hydroxytyrosol (b) to generate ABCA1-enriched cells, which were then incubated with 25 *μ*g/mL of apo-AI for 4 h. Results are expressed as the means ± SEM of at least three independent experiments.

In light of these results, we then investigated the effect of two major phenolic compounds in EVOO-PC (purified tyrosol and hydroxytyrosol) on cholesterol efflux from and ABCA1 protein expression in J774 macrophages. Our results showed that tyrosol and hydroxytyrosol increase in concentration-dependent manner the ABCA1-dependent cholesterol efflux (Figures 4(a) and 4(b), resp.).

## 4. Discussion

Olive oil is the main source of fat in the Mediterranean diet. A large body of knowledge has provided evidence of the benefits of the Mediterranean diet and olive oil consumption on the prevention of atherosclerosis and CHD [24–27]. Several studies have reported that the antiatherogenic effect of olive oil is related to the antioxidant and anti-inflammatory effects exerted by various components, especially monounsaturated fatty acids (MUFA) and polyphenols [11, 17, 28–30]. Phenolic compounds, especially hydroxytyrosol and oleuropein, dose-dependently inhibit LDL and HDL oxidation* in vitro* and* in vivo*, repress superoxide-driven reactions, and break the chain-like propagation of lipid peroxides [31–34]. Interestingly, a study by Covas et al. [17] showed that consuming EVOO increases the postprandial concentration of phenolic compounds in the plasma and in LDL and HDL, which may explain the protective effect of phenolic compounds.

Plasma HDL-cholesterol levels are markedly and inversely correlated to the risk of atherosclerotic cardiovascular diseases [35]. It has been suggested that HDL facilitates cholesterol efflux from peripheral tissues and transports it back to the liver in a process called RCT [36]. ABCA1 facilitates cholesterol efflux from cells to lipid-poor apo-AI but not to HDL [7, 37], whereas another ABC transporter, ABCG1, as well as the SR-BI receptor, is involved in cholesterol efflux from macrophages to HDL [38, 39]. Some studies have suggested that food nutrients and diet may play pivotal roles in the regulation of RCT [25, 40–42]. We have previously shown that EVOO consumption improves the RCT process by enhancing the capacity of HDL to mediate cholesterol efflux and of human monocyte-derived macrophages (HMDM) to excrete free cholesterol [43]. In the present study, we investigated how the consumption of EVOO may promote cholesterol efflux. We focused on the effect of EVOO-PC, especially essential phenols such as tyrosol and hydroxytyrosol.

Our results showed that enriching LDL and HDL with EVOO-PC results in an increase in the resistance of LDL and HDL to lipid peroxidation. This effect may be due to the antioxidant effect of the phenolic compounds, which may scavenge reactive oxygen species and thus inhibit lipoprotein oxidation [16, 44, 45]. Incubating plasma with EVOO-PC increased the binding of polyphenols to LDL and HDL lipoproteins, as previously reported by Covas et al. [17, 46] and Lamucla-Raventós et al. [47]. Moreover, in a recent study, Hernáez et al. [48] showed that olive oil polyphenols increase the size of HDL particles, enhance the stability of HDL by generating a triglyceride-poor core, and enhance the antioxidant status of HDL by increasing the olive oil polyphenol metabolite content of the lipoprotein. Olive oil polyphenols are highly bioavailable, which provides further support for their putative health-promoting effects (reviewed in [49, 50]). However, very few studies have been conducted on the effect of phenolic compounds on RCT. Our results showed that EVOO-PC-enriched HDL promotes RCT by enhancing cholesterol efflux from THP-1-derived macrophages. This effect may be related to an improvement in the physicochemical properties of HDL by increasing their phenol content, which protects HDL from oxidation, and by increasing the fluidity of the phospholipidic layer. Indeed, we previously showed that polyphenol compounds from argan oil (a polyphenol-rich vegetable oil) also enhance HDL-mediated cholesterol efflux by improving HDL fluidity and increasing HDL binding to cell membranes [16]. In the present study, we also investigated the effect of EVOO-PC on cholesterol efflux from THP-1-derived macrophages stressed by Fe/Asc, which induces lipid peroxidation [51] and reduces cholesterol efflux. Pretreating macrophages with EVOO-PC before incubating them with Fe/Asc significantly restored cholesterol efflux from macrophages to HDL, likely by suppressing the effect of Fe/Asc on the cell surface receptors involved in this process. This effect has also been reported with vitamin E and butylhydroxytoluene (BHT), two other antioxidants [23].

However, little is known about the molecular mechanism by which phenolic compounds promote cholesterol efflux. To better understand the mechanism by which EVOO-PC enhances HDL-mediated cholesterol efflux, we investigated the effect of EVOO-PC on cell signaling pathways. Our results clearly showed that EVOO-PC, including tyrosol and hydroxytyrosol, stimulates ABCA1 protein expression in J774 macrophages, which may explain how these phenols promote cholesterol efflux to apoA-1. Uto-Kondo et al. [40] reported that coffee consumption by healthy humans enhances HDL-mediated cholesterol efflux by increasing ABCG1 and SR-BI but not ABCA1 expression and that this may be due to the phenolic acids in the coffee. This appears to be unlikely given that phenolic acids activate liver X receptor-*α* (LXR*α*) expression, which in turn transactivates both ABCA1 and ABCG1. However, other studies, including ours, have shown that resveratrol stimulates LXR*α*, ABCA1, and ABCG1 [52, 53]. It thus appears that different phenolic compounds may stimulate cholesterol efflux via different mechanisms.

In conclusion, our results showed that EVOO-PC enhances the antiatherogenic properties of HDL by reducing oxidative modifications to HDL and by maintaining the physicochemical properties of HDL, which in turn improve the functionality of HDL, especially the capacity to promote cholesterol efflux. EVOO-PC also protected cells from oxidative damage and stimulated ABCA1 protein expression, a key factor in cholesterol efflux and HDL genesis. Our results are in agreement with our previous findings showing that the consumption of olive oil polyphenols helps to reduce cardiovascular risk.

## Figures and Tables

**Table 1:** Clinical and biochemical parameters of participants.

	Mean ± esm
*n* = 24 (w/m)	14/10
Age (mean ± SD years)	30.92 ± 2.55
Body mass index (kg/m^2^)	23.7 ± 1.65
System blood pressure (mmHg)	127 ± 4.65
Dias. blood pressure (mmHg)	78.23 ± 2.09
Total cholesterol (mmol/L)	5.06 ± 0.2
Triglycerides (mmol/L)	1.32 ± 0.15
HDL-c (mmol/L)	1.42 ± 0.09
LDL-c (mmol/L)	3.05 ± 0.15
Apo A1 (g/L)	1.56 ± 0.05
Apo B (g/L)	0.90 ± 0.04
Apo B/Apo A1	0.8 ± 0.04
TC/HDL-c	3.81 ± 0.23
LDL-c/HDL-c	2.5 ± 0.2
TG/HDL-c	1.08 ± 0.17
Glucose (mmol/L)	4.43 ± 0.10
Insulin (pmol/L)	38.32 ± 5.26
CRP (mg/L)	3.16 ± 0.13

TC (total cholesterol); HDL-C (HDL-cholesterol); LDL-C (LDL-cholesterol); CRP (C-reactive protein).
